# "Who owns your poop?": insights regarding the intersection of human microbiome research and the ELSI aspects of biobanking and related studies

**DOI:** 10.1186/1755-8794-4-72

**Published:** 2011-10-07

**Authors:** Alice K Hawkins, Kieran C O'Doherty

**Affiliations:** 1 Centre for Applied Ethics, University of British Columbia, Vancouver, BC, V6T 1Z2, Canada; 2 Department of Psychology, University of Guelph, Guelph, ON, N1G 2W1, Canada

**Keywords:** human microbiome, health research, consent, privacy, ownership, return of results, policy, biobanks, ELSI, research ethics

## Abstract

**Background:**

While the social, ethical, and legal implications of biobanking and large scale data sharing are already complicated enough, they may be further compounded by research on the human microbiome.

**Discussion:**

The human microbiome is the entire complement of microorganisms that exists in and on every human body. Currently most biobanks focus primarily on human tissues and/or associated data (e.g. health records). Accordingly, most discussions in the social sciences and humanities on these issues are focused (appropriately so) on the implications of biobanks and sharing data derived from human tissues. However, rapid advances in human microbiome research involve collecting large amounts of data on microorganisms that exist in symbiotic relationships with the human body. Currently it is not clear whether these microorganisms should be considered part of or separate from the human body. Arguments can be made for both, but ultimately it seems that the dichotomy of human versus non-human and self versus non-self inevitably breaks down in this context. This situation has the potential to add further complications to debates on biobanking.

**Summary:**

In this paper, we revisit some of the core problem areas of privacy, consent, ownership, return of results, governance, and benefit sharing, and consider how they might be impacted upon by human microbiome research. Some of the issues discussed also have relevance to other forms of microbial research. Discussion of these themes is guided by conceptual analysis of microbiome research and interviews with leading Canadian scientists in the field.

## Background

Biobanks, loosely defined as large collections of biological tissues samples, often with some degree of linked clinical or medical information, have received considerable attention in the ELSI (ethical, legal, social issues of genome research) and scientific literature in recent years[1-3]. This attention is due not just to the complexity of the issues raised by biobank related research, but also because they call into question established research ethics norms and accepted practices[4]. The nature of these ethical conundrums has been well documented, with research focused on issues such as privacy, informed consent, ownership of samples and information, secondary use of biological specimens, benefit sharing and governance [5-8]. While these biobank issues remain problematic and unresolved, other areas of science are moving ahead rapidly, and have the potential to further complicate matters[9]. In particular, this paper considers recent large scale research efforts towards studying the human microbiome, and the potential social and ethical implications of this research for biobanks.

Humans can be thought of as a complex of both microbial and human cells, a 'super-organism' [10] containing over 100 trillion microbiota that are essential for nutrition, immunity and pathogen resistance[11]. Whilst some are harmful, the majority of human cell and microbial cell interactions are mutually beneficial, and essential to human physiologic functions and day to day activities[12]. Research on the human microbiome aims to elucidate the relationship between human health, physiology, and behaviour and the various microbial communities present in different areas of our body (including the mucosa, gastrointestinal tract, urogenital system and skin)[13,14]. The goal of the Human Microbiome Project (HMP), established in 2007, is to characterize the role of microbiota in human health and disease. More specifically, the HMP investigates such basic science questions as whether humans share a common core microbiome and whether particular changes in the human microbiome lead to changes in human health and disease states (http://nihroadmap.nih.gov/hmp/)[15]. It is hoped that this research will lead to benefits such as: a better understanding of human nutritional requirements (including how individuals will respond to specific diets), resulting in innovative food production and distribution strategies[10,16,17] and other public health benefits[18,19]; increased knowledge of areas amenable to microbial transplantation and successful manipulation[11]; forensic tools[20,21]; and pharmaceutical improvements known as 'pharmacomicrobiomics'[17,22].

Human microbiome research, in and of itself, raises a number of ethical, legal and social considerations[23]. However, the specific focus of this paper is how these concerns overlap with those debated in the biobank literature and we therefore confine our discussion to issues relevant to this particular context. The types of information and samples collected by different biobanks vary, but with increasing research efforts pertaining to the human microbiome, it is inevitable that an increasing number of biobanks will include collection of specimens required for genomic studies of microbiota associated with humans. To date there has been little attention as to how human microbiome research may affect or further compound the issues debated in the context of biobanks. It is the purpose of this paper to begin to investigate and discuss some of these questions.

Our discussion is informed by both the scientific literature on human microbiome research and the ELSI literature on biobanks. Most importantly, though, we draw on a series of 45 in-depth interviews that were conducted by K.O. in 2009 to 2011. These semi-structured interviews consisted of a series of open-ended questions on the topic of the social and ethical implications of the HMP, and were conducted either in-person, or over the phone. Interview participants were researchers working on the Canadian Microbiome Initiative, and were invited to participate based on satisfying any of three criteria: (i) currently funded to conduct research on the human microbiome; (ii) attendance at a meeting hosted by the Canadian Institutes of Health Research (CIHR) in 2008, the purpose of which was to discuss the form funding for the Canadian Microbiome Initiative would take; (iii) referral based on expertise in a particular aspect of research relevant to the social and ethical aspects of the HMP. Taken together, interviewees represented multidisciplinary expertise on the range of body sites being targeted for study in the HMP, and a spectrum of basic and applied researchers as well as clinicians. The content of individual interviews reflected the particular expertise of the interviewee, and included the subject areas described in the discussion below. Where possible, the discussion that follows is based on peer-reviewed published literature, often suggested by interviewees. Where research is novel or in progress, we rely on anonymized statements of our expert informants to illustrate specific arguments made in the discussion. It is important to note that some of the ethical issues we raise here are relevant to research other than biobank studies. Conversely, there are many other ethical issues related to human microbiome research that are beyond the scope of this paper. We therefore limit our discussion to those ethical issues raised by human microbiome research that intersect with biobank related issues.

Biobank discussions gravitate toward four issues that are now well recognized in the ELSI literature on biobanks: privacy, consent, ownership, and return of research results. Below, we discuss each of these issues in turn, beginning with a brief outline of the problem as currently recognised in the ELSI literature on biobanks, followed by a discussion of the potential implications of human microbiome research on the problem. In this discussion, we pay particular attention to how the social and ethical aspects of biobanks may change or become further compounded as a consequence of microbiome research being conducted using biobank type platforms (see Table 1 for a summary of the issues and concerns raised by biobanks relative to new issues introduce by human microbiome research). We conclude with some recommendations for policy and a consideration of implications for benefit sharing and governance in this evolving field.

**Table 1:** ELSI Issues Raised by Microbiome Research relative to Biobank and Related Studies

ELSI Consideration	Nature of concern	New issues introduced by HMB research
Privacy and Confidentiality	- Discrimination and stigmatisation	- Increased scope of disease predisposition testing - Microbial fingerprints - Potential knowledge of past exposures
	• *For example, predisposition and susceptibility testing*	

Consent	- Respecting autonomy - Invasiveness of sample collection	- Cultural and personal acceptability of research
	• *E.g., research participation of minors (e.g. newborn blood spot collection) *	- *E.g., Vaginal and fecal sample collection*

Ownership	- Human dignity - Benefit sharing	- Samples traditionally considered waste, such as fecal matter
	• *E.g., ownership of blood/tissue samples and cell lines*	- E.g., fecal transplant

Return of Results	- Clinical validity - 'right to know' versus 'right not to know'	- Additional treatment and screening possibilities

Biobank Governance	- Public trust and consideration of societal viewpoints	- Infectious disease
	• *E.g., ensuring minority viewpoints are considered*	

Justice	- Resource allocation	- Global health
	• *For example, payment and health insurance coverage of genetic tests and pharmacogenomics*	

## Discussion

### Privacy

The ability to protect both clinical and genetic data stored in biobanks has been pivotal to biobank discussion since such research was conceived[24]. Due to the potentially sensitive nature of such data, there has been considerable anxiety regarding the possibility of privacy breaches, resulting in personal information being misused[25]. This is of particular relevance in the context of genetic data, as access to an individual's biological specimens and DNA may reveal sensitive information such as predispositions to certain diseases, as well as identity and ethnic background[26]. In spite of increasing recognition that absolute guarantees of privacy protection can no longer be made[27,28], biobanks that have been constructed in ethically sustainable ways go to considerable effort to ensure that personal data is adequately protected to avoid potential discrimination or other adverse events[29].

These concerns are further compounded given recent advances in human microbiome research such as that 'microbial fingerprints' have been found to be potentially as individual as DNA or actual fingerprints. More specifically, the composition of bacterial communities associated with human skin have been found to be unique to each individual, allowing for identification of individuals through analysis of residual skin bacteria recovered from an object (e.g. keyboard or mouse) touched by that person[21]. The identification of an individual by their microbial fingerprint potentially raises privacy concerns similar to those already recognised in genetic and biobank related research. However, the privacy concerns in microbiome research may be somewhat more complex, primarily due to our limited understanding of the relevance of such information at the current time. For example, microbiome technology may allow access to information such as past exposures, or locations an individual has visited in the past (interview 132). As such information is not available from analysis of human DNA alone, this may constitute a very powerful new analytical dimension for use in forensic investigations or by law enforcement or homeland security agencies. Such possibilities promise to be highly controversial given the tensions that already exist between safe-guarding the integrity of health research databases versus pressure from law enforcement agencies to permit greater powers in the use of DNA technologies and databases. Further confounding this, in contrast to DNA, the stability of microbial fingerprints over time is unknown, so it is not possible to say whether such information may be linked to a specific individual for an indefinite period of time (interview 142 & 131).

From a clinical perspective, human microbiome research is still in its infancy, and it will take some time to fully understand the meaning of microbiome findings both on their own, and in the context of other relevant genetic and health information. Similar to concerns with the clinical validity of genome wide association studies[30], there is concern that microbiome research data may be prematurely or incorrectly interpreted. Similar to human genetic information, microbial data may be used, correctly or incorrectly, to reveal an individual's predisposition to certain conditions such as obesity[31]. One can conjecture that human microbiome data even has the potential to reveal an individual's socioeconomic background. Perhaps more significantly, microbiome information, when used in conjunction with genetic information, may tell us a significant amount about a certain individual's susceptibility and predispositions and would be more personally identifying than genetic data alone[11]. For example, asthma is known to be a multifactorial condition, with both genetic and environmental causative factors[32]. Similarly, a typical microbial signature has been identified for inflammatory bowel disease (albeit with a non specific association) (interview 135). A future doctor's visit might therefore conceivably involve both analysis of an individual's human genomic and microbial genomic profile[33]. Knowledge of both would potentially enable greater predictive value than genetics alone of what conditions may develop in an individual's lifetime. Just as with genetic data there are fears of discrimination based on life expectancy or job prospects, for example by employers or insurance companies, and one can conjecture that such microbiome-based discrimination may be more disturbing, as there is potential for discrimination or stigmatisation based on socio economic status, where an individual was born and raised, and which countries an individual has visited[23]. More worrying still, given that we do not know what degree and depth of personal information might be revealed through human microbial analysis, it has been suggested that in some societies such discrimination may even occur on supposed class or caste basis (interview 132). On the other hand, it is also possible that microbiome research may help to counter stigma in some groups. For example, the identification of a microbial predisposition to obesity[34] may lead to greater acceptance and understanding of individuals with obesity. However, it is quite unclear at this point in time in which direction broader public and institutional understandings will develop.

### Consent

The issue of consent in biobank research has been well characterized and discussed. The crux of the concern relates to the ability of a potential research participant to give truly informed consent for a research project in which potential outcomes and effects are unknown, and when the precise nature of future research to be conducted may not yet be conceived[35-39]. Many of these concerns remain similar in the context of microbiome research, where individuals may be asked to give specimens to be deposited in research banks for an indefinite period of time and for an undefined or loosely defined purpose. This raises questions regarding the integrity of the informed consent procedure, potentially leading to the erosion of research subjects' protection and an individual's autonomy in decision making about research participation[6,40]. Biobank debates have suggested a number of proposed solutions to this issue, including broad consent, requiring re-consenting on a regular basis, and adaptive governance mechanisms that allow for representative decision-making on behalf of large numbers of biobank research participants [41,42].

Concerns about consent, as they relate to biobanks, have important implications for microbiome research, both in terms of outlining potential challenges, and in suggesting possible resolutions such as those outlined above. An area in which this is particularly notable is that of child involvement in health research, with questions being raised as to the ethics of children becoming research subjects when they cannot give informed consent[43-46]; for example, infant stool sample collection for microbial research raises similar concerns to new born blood spot collection in genetic biobank research. Moreover, concerns regarding data-sharing may be similar for both microbial and human specimens, in that once samples are shared with other researchers it becomes difficult to regulate or withdraw consent. However, it is important to note that it is possible that microbial research may not be any more ethically problematic than genetic research if microbial data are not stable over time. There is currently scientific uncertainty and disagreement as to the relative stability of an individual's microbiome over time and the degree to which an individual's human genome predetermines their microbiome. If it turns out that there is significant variation over a life-span, and/or a low degree of genetic pre-determination then, for example, a child whose microbial sample was collected in neonatal life may not be identifiable from the specimen over the long term.

In considering current biobanking practices and the potential compounding of ethical implications owing to microbiome research, an important factor that needs to be considered relates to the individual subjective and cultural acceptability of the actual research. For example, biobank research often involves use of a point in time biological specimen, such as a pathology related tissue sample, blood draw or buccal swab. Such sample collection is usually relatively benign, painless and may not require any additional procedures other than what is clinically required. However, microbial research may be considered more invasive, or less culturally acceptable by some groups. Most notably, collection of stool samples or vaginal swabs may not be acceptable to some individuals, or in some countries or ethnic groups (interview 139). This is further exacerbated by the possible need for collection from multiple orifices, and multiple collections over time. Certainly the nature of the research needs to be clearly outlined for an individual to give informed consent for a research study. However it is not unreasonable to conjecture that if some types of research are not acceptable to certain groups, these groups may not participate in research studies, resulting in research findings that are relevant to only certain sub-sectors of society. While this concern is not unique to microbial research, it seems that biobank studies that also investigate aspects of the human microbiome require additional sensitivity to maintain public approval, trust and commitment to the research enterprise.

### Ownership

As alluded to above, microbial specimens may be collected from a number of different collection sites including the nose, mouth, gastrointestinal tract, skin, and urogenital tract (http://nihroadmap.nih.gov/hmp/). As with human biological specimen collection, microbial sample collection may be more or less invasive. However, microbiome research differs somewhat in that some microbial samples are generally considered waste (such as dead skin or feces). Interestingly, the question of ownership arises, a topic that has already led to considerable debate within the biobank literature. In particular, there is debate as to whether a biological specimen, such as tissue, a tumour or blood in some way 'belongs' to the individual it came from, at least for a defined period of time. Irrespective of whether one considers tissue that has been removed from an individual to be owned by them at any given time in the future, at the very least current thinking tends towards informed consent being required not only for removal of a biological specimen, but also for its use in research. A case illustrative of the controversy in this context is that of Henrietta Lacks, an African American woman whose tumour cells were used in medical research to create profitable immortal cell lines without her knowledge or consent[47]. This case raises questions not just about the extent of ownership, but also the potential for future benefit sharing. In particular, discrepancies have been pointed out between the large profits made by companies using Lacks' cell lines and the family and descendants of Henrietta Lacks, who in many cases cannot afford health insurance.

The question of ownership may become even more complex with microbial research. This is particularly the case with research on the gastro intestinal microbiome, which relies heavily on the use of fecal samples. In other words, the question of 'who owns your poop?' now becomes relevant. The question is complex not only because feces has traditionally been considered to be waste, but because of the ambiguous relationship between the genomes of commensal bacteria found in fecal matter and human identity. One the one hand, these genomes are clearly not a part of the human genome and so should not be considered in any way a component of 'being human'. On the other hand, the notion that the human genome has co-evolved to its present state with this multitude of bacterial genomes, and that we require this symbiotic relationship for the maintenance of our health, suggests that the particular collection of microbial genomes each of us carries may be almost as personal as our own genome. At present, there is still much scientific uncertainty about the stability of the microbiome over time, so it is unclear whether a microbial 'waste' sample could still be linked to the individual it came from after a certain number of years. It is thus pertinent to ask whether ownership is indeed a relevant consideration if a sample can no longer be linked back to a certain individual, particularly if this sample was considered waste in the first place. To put it another way, if it turns out that a given individual's microbiome changes and evolves over time, does this make the ownership claim more or less valid?

The issue of ownership is important for two main reasons. The first can broadly be described as having to do with human dignity, especially as it relates to cultural identities, and the second with potential consequences of ownership, particularly financial. Regarding the former, several parallels can be drawn with biobank research utilising blood samples. For example, the Nuu-chah-nulth, an indigenous population on Vancouver Island, agreed to blood sample collection for genetic research on rheumatoid arthritis. However, these samples were later used - without the Nuu-chah-nulth's knowledge or consent - for research into their ancestral origin. This incident was controversial not just because informed consent was not obtained, but also because of the cultural importance of blood and sense of ownership for the Nuu-chah-nulth, who felt that their samples had been stolen[48]. Although fecal matter can hardly be described in the way blood has been as the 'most symbolically precious of substances'[49], it is associated with deep psychological and cultural significance in other ways. Collection of vaginal microbiome samples is similarly highly personal and associated with a range of cultural, social, and psychological sensitivities. In short, collection of microbial samples is potentially highly personal in ways that are quite different from the sense of ownership individuals may experience in relation to other tissue samples. Precisely how this sense of ownership will manifest for research utilising microbial samples is currently unknown and should itself be the subject of research sensitive to cultural, religious, and other contexts.

Regarding the issue of material consequences of ownership, the commercial entitlements of ownership become contentious particularly in discussions of technology development (for example drug, software and biotech development) which may lead to economic gain through intellectual property, sales and marketing, patenting and so on. Such concerns do have similarities with ethical issues raised in tissue biobank research, such as the potential for financial goals to drive research direction, patenting that either stimulates or stifles technology and treatment development, and financial benefit sharing (interview 109). Questions about what is proprietary, particularly when considering publicly funded research, raise ethical issues such as justice, fairness and benefit distribution. For example, as derivation of benefit, financial or otherwise, is one consequence of ownership, there is concern that benefits may only be made available to those who can afford to pay. Benefits of the research, in the form of novel or improved treatment modalities or in the form of financial benefit sharing, may not actually reach those who contributed to research or those in greatest need. The issue is particularly relevant in a broader international context and the question of whether and how microbiome research benefits those in developing nations, will need to be considered[50,51].

These issues are not unique to microbiome studies but may manifest rather distinctly in this type of research or its applications. For example, a controversial method for treating certain forms of bowel disease involves the use of fecal transplants, in which a slurry of feces from a healthy donor (usually a close relative of the patient) is transplanted into the patient. These treatments seem to be extremely efficacious and are based on the notion that the microbial populations within the stool of the healthy donor are able to repopulate and colonise the abnormal gut of the patient and thereby affect a cure. The treatment is controversial, however, as the transplant may also introduce any number of pathogens to the patient. It is thus only used in very extreme cases of illness, and most hospitals are reluctant to allow the procedure at all (interview 140 & interview 116). Work is currently underway to characterise the efficacious bacteria in a healthy gut on a genomic level, to isolate them and turn them into a probiotic treatment that does not require the transplant of fecal matter (interview 140). The point here is that (i) there is evident value in the feces of (at least some) individuals, and that (ii) both the treatment, as well as research pertaining to the treatment, are likely to be associated with cultural sensitivities, awkwardness, and possibly embarrassment on the part of patients and research participants. This suggests that serious consideration be given to questions of ownership as they pertain to human microbiome derived samples.

### Return of results

The issue of whether, how, and under what circumstances to return research results to research participants has been subject to considerable scrutiny both from the public and research perspectives[26,52-55]. In biobank and genetic research the question of return of results usually relates to informing research participants about genetic predispositions, particularly where treatment options or preventative strategies may be of value and individuals may act on this information[56]. Some argue that research participants have a right to know such information[57,58], whilst others argue that there is a right not to know[59,60]. Researchers voice concerns that requiring return of results places an unjust burden on them, making the research enterprise untenable[5]. In addition, unless the clinical utility of research results is firmly established by evidence based studies, there is a risk of harm due to the premature or inadequate translation of research results[61,62].

These debates are equally relevant to microbial research, and similar questions are raised, including whether results should be returned on a public or individual level and who is responsible for result disclosure and explanation[23]. It is possible however that return of microbial research results may actually have more impact, on an individual and public level, than genetic research results, if an individual is found to have a potentially harmful microbial profile that is easily treated. There has been much debate about the utility of genetic information, particularly when there is little that can be done to mitigate such genetic risks[63]. Given that commensal microbial populations are likely to be more malleable than human genes, knowledge of an individual's microbial profile may open up far more decisions points for treatment, diagnosis, and risk management than knowledge of one's genome[64]. This has even more profound implications if there is an infectious component to a microbial profile so that return of research results may be of immediate public health interest.

## Summary

As outlined in our introduction, although there are a range of ELSI issues raised by human microbiome research, we have focussed primarily on the intersection of human microbiome research and biobanking. Many of the ELSI issues raised by human microbiome research in this context are not significantly different from those already encountered in human tissue biobank research. However, there are some areas in which the already difficult considerations involving privacy, consent, ownership and return of results are further complicated by the additional collection and study of microbial DNA associated with humans. Identifying these challenges is not intended to hinder microbiome research, but rather to assist researchers, clinicians, and patients to work together in ways that build understanding and cooperation, while avoiding unnecessary conflict. Engaging with these challenges at a relatively early stage of the science also allows meaningful and relevant policy guidelines to be formulated for human microbiome research and its applications in a proactive rather than reactive manner.

### Governance considerations

In considering the rather specific issues of privacy, consent, return of results, and ownership in the context of research ethics and biobanks, there has been growing recognition first, that these issues cannot be resolved in isolation from each other and, second, that their resolution must involve a strong focus on governance. While operationalization of standardised approaches to biobank governance are only just beginning to emerge, current thinking on the subject tends to emphasise the need to facilitate health research while enhancing the capacity for participant and public involvement as well as benefit sharing[44,65]. This trend is likely to continue with research endeavours relating to the human microbiome, but will inevitably require creative adaptation to apply to novel circumstances introduced by microbial research. Moreover, while many human genome researchers have become accustomed to thinking about the social and ethical implications associated with their research, microbial researchers may not have previously engaged with such issues as the need to protect research participants' privacy, and dealing with incidental findings such as disease predispositions and whether (and how) to communicate these results back to participants.

Considerations about governance may become even more pressing and complex with the advent of human microbiome research due to the public health implications of such research. For instance, while already complicated, considerations about biobank governance have generally not had to take into account issues such as infectious disease. This is clearly an important factor for microbial research, and mechanisms for interfacing research platforms with public health agencies will likely need to be designed. Importantly, this link will need to be made transparent to research participants to conform with adequate informed consent criteria.

### Justice considerations

In addition, as alluded to in our discussion on ownership and return of results, provisions for benefit sharing are relevant in this context[50]. The precise form that benefit sharing might take can vary, but involves ensuring that the results of research are distributed with some sense of justice and fairness. Benefits of research can be conceptualised in terms of financial returns, medical technologies, or public health benefits, while appropriate beneficiaries might include the researchers, research participants, funders, identifiable minorities and the wider public, depending on context. Similar to the charges of bioprospecting and biopiracy that have been levelled against some genetic researchers for exploiting indigenous groups without appropriate compensation, microbiome research may lend itself to exploitation of groups and individuals for the benefit of already privileged segments of the population. For example, given the importance that is attributed to commensal bacteria in human health, research is likely to focus on producing probiotic interventions through studying correlations between particular health variables (e.g., cancer resistance, immunity, women's health) and the microbial profiles of certain individuals or groups. Without appropriate governance, the primary health benefit of these probiotics will flow to those who can afford them, while the associated financial benefits will flow to researchers and developers. While there can be no a priori objection to this, there is a sense of incongruity if the research participants who contributed time, samples, and exposed themselves to the potential risks of research involvement share neither financial nor health benefits.

### Recommendations

In conclusion, our consideration of the social and ethical dimensions of the intersection of human microbiome research and tissue biobanking suggest some recommendations for policy:

1. We need to be mindful of, and protect against, novel and unanticipated forms of discrimination that may arise from microbiome research. Such discrimination may come in many forms, and be based on any number of levels of categorization including socio-economic factors, and cultural or ethnic background (interview 137). Marginalised sectors of society who already suffer from discrimination, such as minorities and those with obesity, may find that microbiome research leads to a greater 'scientific' basis for marginalisation - the results of which could be abhorrent. Also, association of diseases such as cancer with a microbial aetiology (whether correct or incorrect) may lead to stigmatisation of groups reminiscent of those associated with certain infectious diseases.

2. As with other forms of biomedical research, microbiome research needs to be sensitive toward the socio-cultural and economic context of its research participants. Given the nature of microbial research on samples sourced from humans, particular attention should be given to respecting the sensitivities of participants. Success in this regard is likely to be rewarded with strong research participation, and stronger relationships of trust between scientists and the larger public. Failure will inevitably lead towards further alienation between the scientific establishment and certain members and subgroups of the public.

3. As outlined above, microbial data may be as powerful as genetic information in calculating predispositions to disease. In addition, knowledge of the interactions between human and microbial genomes will certainly provide a far superior platform for calculating disease predispositions. Microbiome research studies will need to take this into account and make adequate provision for making decisions to withhold or disseminate such information to research participants.

4. Until there is evidence to the contrary, information about an individual's microbiome should be treated with the same safeguards as human genetic information. This means that biobanks (whether they hold biological samples and/or information) need to make adequate provision for data storage protection mechanisms to diminish potential privacy breaches. In this context, the concept of what is truly 'personal' information needs further exploration and definition. As with other forms of data, privacy breaches of microbial data have the potential for harmful effects, infringement of autonomy, stigmatization and discrimination, yet these need to be balanced with public health considerations. Awareness of these potential outcomes should enable us to build in adequate privacy protections at the beginning of such research endeavours. Anonymization of specimens may be an option, however may be meaningless if the sample can be linked back to an identifiable sample elsewhere[27]. In addition, anonymization of specimens may be less fruitful for the research endeavor[42].

5. Given that many researchers working on the HMP have not had previous experience with human subjects research, efforts might be made to increase awareness among researchers about the social and ethical implications associated with such research.

6. Finally, the question of 'who owns your poop?' will finally need to get the attention it really deserves. If only to avert costly court cases and mismatched expectations between researchers and participants, a reconsideration of the categorisation of human waste may be important.

## Competing interests

The authors declare that they have no competing interests.

## Authors' contributions

KO conceived of the study, carried out the background research and conducted the interviews for the study. AH conducted interview analysis for the study. AH and KO coordinated and drafted the manuscript. All authors read and approved the final manuscript.

## Pre-publication history

The pre-publication history for this paper can be accessed here:

http://www.biomedcentral.com/1755-8794/4/72/prepub
